# T cell senescence by N-glycan branching

**DOI:** 10.18632/aging.204239

**Published:** 2022-08-13

**Authors:** Haik Mkhikian, Michael Demetriou

**Affiliations:** 1 Department of Pathology and Laboratory Medicine, University of California, Irvine, Irvine, CA 92697, USA; 2 Department of Microbiology and Molecular Genetics, University of California, Irvine, Irvine, CA 92697, USA; 3 Department of Neurology, University of California, Irvine, Irvine, CA 92697, USA

**Keywords:** T cell, immune-senescence, N-glycan branching, N-acetylglucosamine, interleukin-7

Immune-senescence refers to the aging of the immune system and the resulting deterioration in its ability to fight infections and cancer in older individuals. This phenomenon is reflected in the numerous infectious diseases which show increased severity in the elderly, most recently demonstrated by the COVID-19 pandemic. The trajectory of the COVID-19 pandemic would have differed markedly if the severity of disease in older individuals was similar to healthy young individuals. Therefore, understanding what factors contribute to immune-senescence and how to reverse them is of great importance.

While several defects are thought to contribute to immune-senescence, the molecular mechanisms involved remain poorly understood. In recent work we discovered that altered glycosylation in T cells contribute to T cell aging [1]. In particular, the level of N-acetylglucosamine (GlcNAc) branching on cell-surface N-glycans was increased in T cells, most prominently in naïve CD4^+^ T (T_N_) cells, from old mice and humans. Surprisingly, we found that old females had a greater age-dependent increase in branching than males. Our previous work has demonstrated that N-glycan branching is a critical negative regulator of T cell activation and pro-inflammatory responses [2,3], with decreased branching leading to hyperactivity, inflammation and autoimmunity. Thus, the elevated branching observed with aging is expected to result in T cell hypoactivity and contribute to T cell aging. Indeed, reversing this elevation in N-glycan branching rejuvenated old CD4^+^ T_N_ responses *in vitro* and resulted in reduced disease severity in an *in vivo* model of salmonella infection in old female mice.

Mechanistic studies revealed that the elevated branching was driven by cell-extrinsic factors in old mice, with further investigation identifying that elevated interleukin-7 (IL-7) signaling in old CD4^+^ T_N_ cells was the major driver of branching in mice. In humans, serum levels of the simple sugar N-acetylglucosamine (GlcNAc), a key N-glycan branching pathway metabolite whose serum deficiency is associated with severity of the autoimmune disease multiple sclerosis and reduced myelination [4,5], were also elevated in an age dependent manner. Interestingly, although serum GlcNAc was elevated in both sexes, it only associated with T_N_ cell branching in females, suggesting another factor may be involved. Indeed, although supplementation with GlcNAc alone has been shown to raise branching in activated and proliferating T cells, it did not raise branching in resting human CD4^+^ T_N_ cells. However, the combination of IL-7 and GlcNAc synergistically raised branching in CD4^+^ T_N_ cells, suggesting that similar to mice, elevated IL-7 signaling in females may explain the sex differences in branching.

These studies point to several potential therapeutic targets, including the N-glycan branching pathway, IL-7 signaling, and serum GlcNAc levels. These can be separated into short-term interventions, such as during vaccination or illness, versus long-term rejuvenation for prophylaxis. Although we showed that blocking IL-7 signaling in old mice can reverse elevated branching in old CD4^+^ T_N_ cells, IL-7 is thought to be critical for homeostatic maintenance of the T cell pool. Thus, blocking IL-7 alone is unlikely to be effective. However, combining IL-7 agonism with inhibitors of N-glycan branching, thereby blocking the IL-7 dependent increase in branching, may provide the best of both worlds, namely maintenance of a T cell pool that remains potent in triggering immune responses. N-glycan branching inhibitors in cancer trials in humans displayed acceptable safety with branching reduced >50% in leukocytes [6–8]. As only a small reduction in branching (~20%) is required to rejuvenate old T cells, low dose inhibition of branching should be safe, particularly for short-term immune boosting.

An alternative approach would be to target elevated GlcNAc. Although currently unexplored, this is in some ways the simplest and perhaps best therapeutic avenue. Therapeutically reducing GlcNAc to “young” levels may be the least biologically invasive approach. Though no targeted methods exist, further studies may reveal the mechanism for elevated serum GlcNAc with age, thereby providing potential novel therapeutic targets. GlcNAc is largely uncatabolized by mammalian cells and is excreted by the kidney; therefore levels may accumulate with lifelong exposure of dietary GlcNAc and/or declining kidney function with age. Alternatively, elevated production or a leaky cellular source may be responsible for the elevation.

Another interesting question that remains unknown is what gives rise to the sex difference in IL-7 signaling. Interestingly, IL-7 signaling is elevated specifically in CD4^+^ T_N_ cells, and not CD4^+^ memory cells, suggesting a more complex mechanism than systemic elevations of IL-7. Intriguing possibilities include perturbations to lymphoid organ architecture and specific alterations to a naïve T cell niche. Regardless, ovariectomy with or without thymectomy was insufficient to increase branching, suggesting that the age associated decrease in ovarian female sex hormones is inadequate. However, from these experiments it is not clear whether ovarian hormones are uninvolved or necessary but insufficient. Reconstituting ovarian hormones in old female mice by supplementation of specific hormones or transplantation of young ovaries would help distinguish these possibilities. Importantly, identifying such a mechanism could provide an additional therapeutic avenue, such as estrogen supplementation in older humans.

Finally, our findings may also aid in differentiation of healthy aging from immune-senescence. Serum GlcNAc and/or CD4^+^ T_N_ cell N-glycan branching levels may serve as useful biomarkers of T cell aging. In future work it will be important to determine whether variability in serum GlcNAc and/or N-glycan branching correlates with variability in immune function or vaccine responses in the elderly. Indeed, such a biomarker could help distinguish older individuals at highest risk of severe infections and prioritize them for treatment or vaccination.
